# HOXB1 restored expression promotes apoptosis and differentiation in the HL60 leukemic cell line

**DOI:** 10.1186/1475-2867-13-101

**Published:** 2013-10-22

**Authors:** Marina Petrini, Federica Felicetti, Lisabianca Bottero, Maria Cristina Errico, Ornella Morsilli, Alessandra Boe, Alessandra De Feo, Alessandra Carè

**Affiliations:** 1 Department of Hematology, Oncology and Molecular Medicine, Istituto Superiore Sanità, Rome 00161, Italy

**Keywords:** HOXB1, AML, Apoptosis, Gene expression, Promoter methylation

## Abstract

**Background:**

Homeobox (HOX) genes deregulation has been largely implicated in the development of human leukemia. Among the HOXB cluster, HOXB1 was silent in a number of analyzed acute myeloid leukemia (AML) primary cells and cell lines, whereas it was expressed in normal terminally differentiated peripheral blood cells.

**Methods:**

We evaluated the biological effects and the transcriptome changes determined by the retroviral transduction of HOXB1 in the human promyelocytic cell line HL60.

**Results:**

Our results suggest that the enforced expression of HOXB1 reduces cell growth proliferation, inducing apoptosis and cell differentiation along the monocytic and granulocytic lineages. Accordingly, gene expression analysis showed the HOXB1-dependent down-regulation of some tumor promoting genes, paralleled by the up-regulation of apoptosis- and differentiation-related genes, thus supporting a tumor suppressor role for HOXB1 in AML. Finally, we indicated HOXB1 promoter hypermethylation as a mechanism responsible for HOXB1 silencing.

**Conclusions:**

We propose HOXB1 as an additional member of the HOX family with tumour suppressor properties suggesting a HOXB1/ATRA combination as a possible future therapeutic strategy in AML.

## Background

HOX genes form a subset of the larger family of homeobox genes [1], encoding transcription factors with a conserved 60 amino-acid, helix-turn-helix DNA-binding domain, known as homeodomain. Human HOX genes are organized on different chromosomes in four clusters A, B, C and D, consisting of nine to twelve tandem genes [2]. Although firstly identified as morphogenetic regulators during embryonic development [3], many evidences have shown that HOX containing genes play also a significant role in normal and leukemic haematopoiesis [4]. In particular, in primitive CD34^+^ populations HOXB cluster genes are coordinately transcribed during differentiation of myeloid, erythroid [5,6] and lymphoid cells [7]. Also some HOXB genes have been associated with specific functions and stages of the hematopoietic maturation: overexpression of HOXB4 has been shown to favour self-renewal of more primitive populations over differentiation [8], whereas HOXB6 expression is required for normal granulo- and monocytopoiesis and its deregulation associated with a maturation block [9]. HOX genes as HOXA9, HOXC11 and HOXD13 have been implicated in chromosomal translocations associated with myeloid leukemia where they are fused with the nucleoporin gene NUP98 [10]. Expression profiles of pediatric AMLs obtained by Real-time PCR arrays revealed a novel signature of HOX down regulated genes, including HOXB1 which results significantly repressed (mean values 23.5 in normal controls vs 0,8 in AMLs) [11]. Even so the authors did not discuss its tumor suppressor role. Other HOX genes, as HOXA5 in breast cancer, have been described as tumor suppressor genes [12,13]. In addition HOXA5 loss of expression, due to promoter hypermethylation, has been also suggested to arrest normal differentiation in AML [14]. Recently the first genome-wide survey of the DNA methylome performed in sporadic pituitary adenomas demonstrated the association between increased methylation of HOXB1 and its significantly reduced transcription [15]. In the present study we showed that HOXB1 was expressed in normal lymphocytes, erythrocytes, granulocytes and monocytes as well as in human multipotent CD34+ cells purified from peripheral blood of healthy donors, whereas it was not detectable in a number of analyzed primary AML blasts and leukemic cell lines. The deficiency of HOXB1 in leukemic cells, in contrast with the reported wide spread expression of other HOXB genes in AMLs [16], prompted us to investigate whether its enforced expression could restore any biological function pushing the leukemic blasts towards apoptosis and/or differentiation. Moreover, as it is known that epigenetic deregulation of critical genes can contribute to leukemogenesis [17], we evaluated HOXB1 gene silencing as a consequence of promoter CpG island hypermethylation or histones acetylation in the HL60 cell line. Finally, trying to dissect the molecular pathways possibly triggered by HOXB1, we searched its downstream genes by using an Atlas Human Cancer macroarray.

## Materials and methods

### Cells and cell cultures

The leukemia cell lines, including promyelocytic HL60 and NB4, myeloblastic AML193, monocytic U937, erytroblastic K562 and the lymphoid T cell Peer and CCRF-CEM, were grown in RPMI 1640 medium (Gibco Invitrogen, Grand Island, NY), supplemented with heat-inactivated fetal bovine serum (FBS) (HyClone, Logan, Utah). HL60 cell line was also grown in the presence of differentiation factors: all trans retinoic acid (ATRA) (Sigma-Aldrich, St. Louis, MO) at 10^-7^ M and 1α,25 dihydroxyvitamin (VitD3) (Sigma-Aldrich, St. Louis, MO) at 10^-8^ M, over a period of 7 or 11 days of culture, respectively. When indicated HL60 cells were also treated with Z-Val-Ala-DL-Asp(OMe)-fluoromethylketone (z-VAD) (Bachem, Bubendorf, Switzerland) 25 μM alone or in combination with ATRA. The human teratocarcinoma (NT2D1) cell line, utilized as a positive control of HOXB1 expression, was grown in DMEM medium, 10% FBS supplemented and induced to differentiate by ATRA 10^-7^ M over a period of 9 days.

Cryopreserved cell samples obtained from a group of twelve patients with acute myeloid leukemia were studied and subclassified according to the FAB nomenclature (staged from M1 to M6) and cytogenetic analysis (7CN-AML lacking major translocation, 3INV16 and 2 t:15,17) [see Ref. 9]. The original samples (two for each group) contained a range of 20 to 500×10^6^ cells and >80% of blastic infiltration. Leukocytes were isolated by Ficoll-Hypaque density centrifugation. Normal granulocytes, monocytes/macrophages, lymphocytes and erythroblasts were obtained from peripheral blood of healthy donors. CD34+ progenitor cells were purified from peripheral blood as reported [18].

### Retroviral gene transduction

The HOXB1 cDNA encompassing its complete coding sequence was cloned into the retroviral vector LXSN as LB1SN; the LXSN empty vector was always used as an internal control [19]. AML193, U937, NB4 and HL60 cell lines were transduced with the LXSN empty vector and with LB1SN helper-free virus containing supernatants. Cells were treated twice for 4 hr with undiluted packaging cell supernatants in presence of 8 μg/ml of polybrene. Infected target cells were grown for 48 hr and then selected with G418 (0.8 mg/ml). As the ectopic expression of HOXB1 in AML193, U937 and NB4 cell lines was apparently lost in the first days after selection (see Additional file 1: Figure S1 and not shown), the subsequent functional studies were performed on the sole HL60 cell line.

### RNA analysis

HOXB1 expression was evaluated either by traditional or Real-time RT-PCR. For the traditional technique relative quantifications were done by densitometric analysis after GAPDH samples normalization. When indicated PCR products were verified by southern blotting using an internal probe. Negative samples were confirmed after 40 amplification cycles.

Real-time RT–PCR was performed by the TaqMan technology, using the ABI PRISM 7700 DNA Sequence Detection System (Applied Biosystems, Foster City, CA) as reported [19]. Commercial ready-to-use primers/probe mixes (Assays on Demand Products, Applied Biosystems) are listed: HOXB1: #Hs00157973_m1; early growth response 1 (EGR1): #Hs00152928_m1; fatty acid synthase (FASN): #Hs00188012_m1; mouse double minute 2 homolog (MDM2): #Hs00234760_m1; programmed cell death 10 (PDCD10): #Hs00200578_m1; caspase2 (CASP2): #Hs00154240_m1; non metastatic cells 1 protein (NME1): #Hs00264824_m1; secreted protein acidic and rich in cysteine (SPARC): #Hs00234160_m1, Glyceraldehyde-3-phosphate dehydrogenase (GAPDH) #Hs4326317E.

### cDNA expression array

Commercially available cDNA expression arrays (Atlas Human Cancer cDNA expression array 1.2, containing 1176 human genes involved in cancer, Clontech, Mountain View, CA) were used to compare gene expression of LXSN- and HOXB1-transduced HL60 cell line. Arrays, twice repeated, were screened according to the manufacturer’s protocol and as reported [19]. The gene list of Table 1 was obtained by using 1.6 as cutoff value.

**Table 1:** Differentially expressed genes evaluated by macroarray in HL60/HOXB1 vs HL60/LXSN

**Gene**	**GenBankID**	**Function**	**Ratio HOXB1/LXSN**	**qRT-PCR ratio**
**BRCA2**	U43746	Oncogenes & tumor	0,10	
**CCNI**	D50310	Cell cycle regulators	0,34	
**EGR1**	X52541	Transcriptional regulators	0,21	0,02
**FASN**	S80437	Fatty acid/Lipid metabolism	0,13	0,25
**FBP2**	U69126	Hydrolases/carbohydrate biosynthesis	0,50	
**MDM2**	Z12020	Oncogenes/Apoptosis assoc. proteins	0,31	0,5
**OAS1**	M11810	NucleotidylTransferases	0,06	
**SKY**	D17517	Protein kinases receptors	0,58	
**SOD1**	K000454	Antioxidant/Oxidoreductases	0,65	0,7
**TNFRSF1A**	L41690	Growth factor receptors	0,06	
**AKAP1**	X97335	Mitochondrial targeting of proteins	1,94	
**CASP2**	U13021	Cysteine proteases/Caspases	1,63	1,4
**CCND3**	M92287	Cell cycle regulators	4,54	
**CDC37**	U63131	Cell cycle regulators	7,03	
**CRM1**	Y08614	Transporter proteins	2,43	
**MAPRE1**	U24166	Cytoskeleton Regulators	1,62	
**EIF3B**	U78525	Initiation of translation factors	2,49	
**ERBB3**	M29366	Receptor tyrosine kinases	3,00	
**JNK2**	L31951	Intracellular kinase network members	1,93	
**KPNB1**	L38951	Transporter proteins	1,59	
**NME1**	X17620	Kinases/ Transferases	2,62	1,4
**PDCD10**	AF022385	Apoptosis-associated proteins	3,50	1,4
**PTP4A1**	U48296	Protein tyrosine phosphatases	2,00	
**RPS5**	U14970	Ribosomal proteins	3,00	
**SPARC**	J03040	Matrix-associated proteins	2,85	1,5
**ST13**	U28918	HSC70-interacting proteins	2,00	
**TRAM**	X63679	Secreted protein translocation	2,55	

Differential gene expression was confirmed by RT-PCR when indicated. The reported ratio indicates the observed fold increase or decrease of the HL60/HOXB1 gene expression over HL60/LXSN. Cut-off value 1,6.

### Western Blotting

Protein analysis was performed by immunoblot according to standard procedures. The primary antibodies used were: rabbit polyclonal anti-HOXB1 (Covance Research Products, Berkeley, CA); anti-apoptotic peptidase activating factor 1 (APAF1) and anti-BCL2-associated X protein (BAX) (BD, San Jose, CA); anti-histone deacetylase 4 (HDAC-4) and anti-caspase3 (CASP3) (Cell Signaling Technology, Beverly, MA); anti-B-cell CLL/lymphoma 2 (BCL2) and anti-myeloid cell leukemia1 (MCL-1) (Santa Cruz Biotechnology, Dallas, TX) and mouse monoclonal anti-actin (actin) (Calbiochem, La Jolla, CA).

### In vitro growth and cell cycle assays

The proliferative rate of LXSN- and HOXB1-transduced cells was evaluated by a XTT-based colorimetric assay (Roche Molecular Biochemicals, Mannheim, Germany) [19] and the Trypan-Blue exclusion dye test. Cell cycle analysis was performed using a CycleTEST™ PLUS Kit (BD, San Josè, CA) on HL60 cells, transduced or not with HOXB1.

### Apoptosis assay

For each sample 10^5^ cells were incubated and stained according to standard procedures (TACS™ AnnexinV-FITC apoptosis detection Kit) (R&D Systems Inc, Minneapolis, MN). Results were expressed as total absolute percentages of AnnexinV^+^, Annexin^+^/PI^+^and PI^+^ gated cells.

Apoptosis was also evaluated by the ApoONE Homogenous Caspase 3/7 Assay. A spectrofluorometer 96 wells plate reader (Wallac VICTOR2, Turku, Finland) was used for measuring the fluorescence of 5×10^4^ cells/well of both HL60/LXSN and HL60/HOXB1. Cells were kept in 1% FBS or in 10% FBS. As a control, cells were grown in the presence of staurosporine at 200nM for 1 hr.

### Cell surface markers and morphological analysis

To evaluate the granulocytic and monocytic differentiation capacities, LXSN- and HOXB1- transduced HL60 cells were grown in vitro up to 7 or 11 days in the presence of 10^-7^ M ATRA or 10^-8^ M VitD3, respectively. Cells were then analyzed for cell surface markers and morphology. Specifically, the cells were labelled with anti-CD11b and anti-G-CSF receptor (G-CSFR) (for G-lineage differentiation), double stained with anti-CD14/anti-CD11b (for M-lineage differentiation) (Pharmingen, San Diego, CA) and subjected to FACS analysis (FACS Scan Becton Dickinson, San Diego, CA).

Cell morphology was evaluated on May-Grünwald-Giemsa stained slides according to standard criteria. Classification includes blasts, promonocytes and promyelocytes as intermediate cells, and monocytes, myelocytes and beyond as mature cells. Three separate experiments were analyzed by two independent blind observers.

### Epigenetic analysis of HOXB1 promoter

The methylation status of CpG islands of HOXB1 promoter was evaluated by the SABiosciencesEpiTect Methyl DNA Restriction kit (Qiagen, Gaithersburg, MD) [20]. HOXB1 CpG island location was Chr17:46607804–46608390. Related RefSeq ID: NM_002144 (HOXB1). Briefly, 250 ng of DNA-RNA free, extracted by the DNeasy blood and tissue KIT (Qiagen), were digested in four equal reactions with no enzymes, methylation-sensitive enzyme, methylation-dependent enzyme, or both enzymes according to the manual instructions (EpiTect® Methyl qPCR Assay Handbook, http://www.qiagen.com). To determine the relative amounts of hypermethylated (HM), intermediately methylated (IM) and unmethylated (UM) DNAs, the products of these reactions were amplified by SABiosiences EpiTect Methyl qPCR primer assay for human HOXB1 (MePH22204-2A). To analyze the effects of demethylation on HOXB1 gene expression, we treated HL60 cells (0,5×10^6^/ml) for 1 up to 5 days with the demethylating agent 5-Azacytidine (5-AzaC) at 1 μM and 5 μM concentrations (Sigma-Aldrich, Saint Louis, MO), replacing medium and adding new 5-AzaC every 48 hrs. Moreover, to evaluate HOXB1 epigenetic regulation by the histones acetylation-deacetylation mechanisms, we treated the HL60 cells (0,5×10^6^/ml) with 100 or 600 ng of the histone deacetylase inhibitor Trichostatin A (TSA) (Sigma-Aldrich) for 48 and 72 hr [21]. Following all the above mentioned treatments, we searched for HOXB1 mRNA re-expression in HL60 cells by RT-PCR.

### Statistical analysis

All the experiments were repeated at least three times, unless otherwise stated. Reported values represent mean ± standard errors (S.E). The significance of differences between experimental variables was determined using parametric Student’s t-test with P < 0.05 deemed statistically significant. P-values relative to HOXB1-transduced cells were always referred to LXSN-transduced cells.

## Results

### HOXB1 is downregulated in leukemic cells

We evaluated the endogenous expression of HOXB1 in a panel of representative primary acute myeloid leukemia (AML) cells, staged from M1 to M6, and some stabilized leukemic cell lines (U937, HL60, AML193, NB4, K562, CEM and PEER). As normal controls, we utilized terminally differentiated cells, including granulocytes, monocytes, macrophages, erythroblasts and lymphocytes, as well as CD34+ progenitors from peripheral blood.

**Figure 1:**
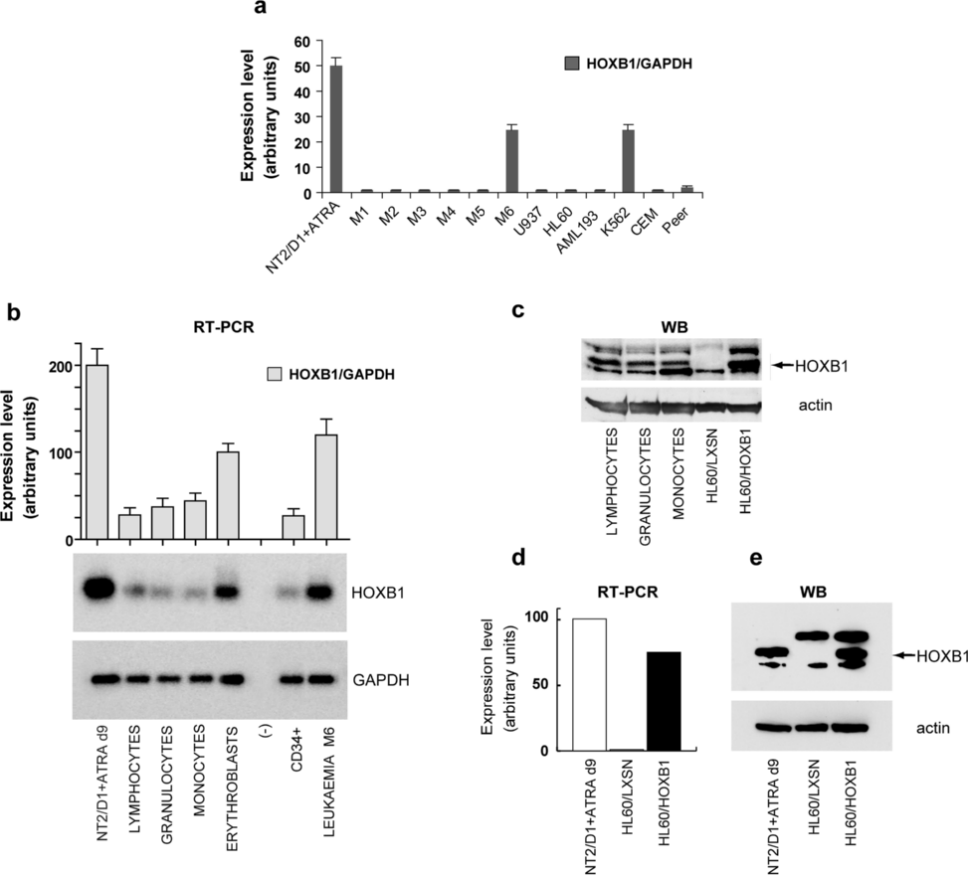
**HOXB1 expression analysis.** Real time RT-PCR in a panel of leukemic cell lines (U937, HL60, AML193, K562, CEM, Peer) and in primary cells from patients staged from M1 to M6 AMLs **(a)**. Representative RT-PCR in lymphocytes, granulocytes, monocytes, erythroblasts and CD34+ from normal peripheral blood, and in M6 erythroleukemia (b, bottom). The relative quantifications are reported as the ratio HOXB1/GAPDH **(b, top)**. HOXB1 protein expression in normal peripheral blood cells compared to HL60/HOXB1 cells **(c)**. Real time RT-PCR in LXSN- and HOXB1-transduced HL60 cell lines **(d)**. Western blot analysis of HOXB1 **(e)**; actin was used for normalization. NT2/D1 cells, treated with ATRA for 9 days, were utilized as positive controls.

As determined by qReal-Time and traditional RT-PCR, HOXB1 was barely or not expressed in all the examined neoplastic cells, even after 40 cycles of amplification (Figure 1a), whereas it was detectable, at RNA and protein levels, in normal cells purified from peripheral blood and in CD34^+^ progenitors (Figure 1b and c). Among the AMLs the exceptions, showing HOXB1 expression, were the M6 staged erythroleukemias and the K562 cell line, possibly in agreement with their predominant erythroblastic cells component (Figure 1a and b). In all the experiments a 9 days ATRA-induced teratocarcinoma NT2/D1 sample was included as a positive control (Figure 1a, b, d and e).

### HOXB1 restored expression induces apoptosis and cell death in HL60

To investigate the functional role of HOXB1, we selected the AML193, U937, NB4 and HL60 cell lines as models for gene transduction. To this end was utilized the retroviral vector LB1SN and the correct transcription and translation of HOXB1 mRNA and protein were confirmed by qReal-Time RT-PCR and Western blot analysis (Figure 1c-d-e, Additional file 1: Figure S1 and not shown). Unfortunately, as the enforced expression of HOXB1 resulted quickly lost in AML193, U937 and NB4, the sole HL60 cell line was exploitable to determine whether HOXB1 overexpression might actually affect the biological properties of HL60 cells.

**Figure 2:**
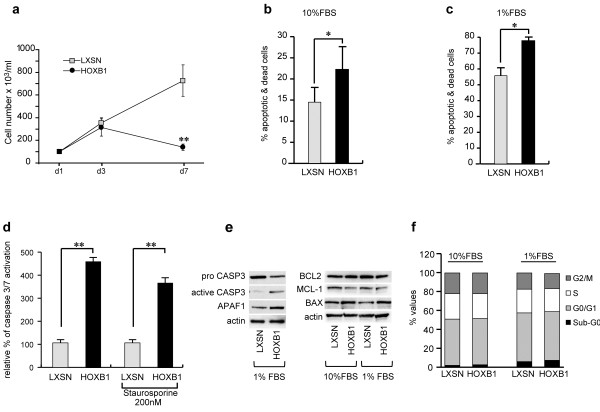
**Effects of HOXB1 restored expression in HL60 cell line.** Analysis of cell growth **(a)** and percentage of apoptotic & dead cells, as evaluated at day 7 of culture by the Annexin/PI analysis system in 10% **(b)** and 1% **(c)** FBS, *p < 0.01. Activation of CASPASE 3/7 in LXSN- and HOXB1-transduced HL60 cells in low serum condition and in presence of 200 nM staurosporine in 10% FBS **(d)**, **p <0.005. Western blot analysis of pro- and active CASPASE 3 and APAF1 **(e left)** and BCL-2, BAX and MCL1 proteins **(e right)**. Actin was used for normalization. Cell cycle analysis in LXSN- and HOXB1-transduced HL60 cells **(f)**.

We then performed some representative *in vitro* functional assays in high (10%) and low (1%) serum conditions. In order to evaluate the proliferative rate, cells were initially seeded at 1×10^5^/ml and monitored up to 7 days when a significant reduction of cell growth (equal to 70%) was visible in HOXB1-expressing cells, regardless of serum concentration (Figure 2a and data not shown). Looking for the cause of such reduction, we compared the total apoptotic rates (including annexin^+^, annexin^+^/PI^+^ and PI^+^ cells) detectable in HOXB1- and LXSN-transduced cells. Interestingly, in HOXB1/HL60 cells we observed an increase from 14% to 22% in high serum (Figure 2b), and an even greater enhancement, from a basal 54% up to 77%, in low serum cell cultures (Figure 2c).

To identify which members were mainly involved in the HOXB1-dependent apoptotic process, we analyzed by western blot a number of apoptosis related factors in HOXB1- vs LXSN- HL60 cells kept in 1% serum condition. Results showing the functional activation of caspase 3&7 (> 4 fold) (Figure 2d) were confirmed by the induction of the cleaved form of CASP3 protein (Figure 2e left). The caspase activating factor, staurosporine (200 nM) was included as a positive control (Figure 2d).

In addition the role of HOXB1 was sustained by the differential expressions of the antiapoptotic Bax and the proapoptotic Mcl1 proteins, respectively induced and downregulated by HOXB1. The Bax/Bcl2 ratio, doubled by HOXB1, was also indicative of a more apoptogenic balance (ratio Bax/Bcl2 0.7 in LXSN- and 1.3 in HOXB1-HL60) (Figure 2e right). Finally, in the HOXB1 expressing cells we observed the upregulation of the proapoptotic factor APAF1 (Figure 2e left).

In view of the lack of significant differences in the cell cycle analysis of HOXB1- respect to LXSN-transduced cells (Figure 2f), we could consider the apoptotic process as the main mechanism underlying the HOXB1-dependent decrease of cell growth.

**Figure 3:**
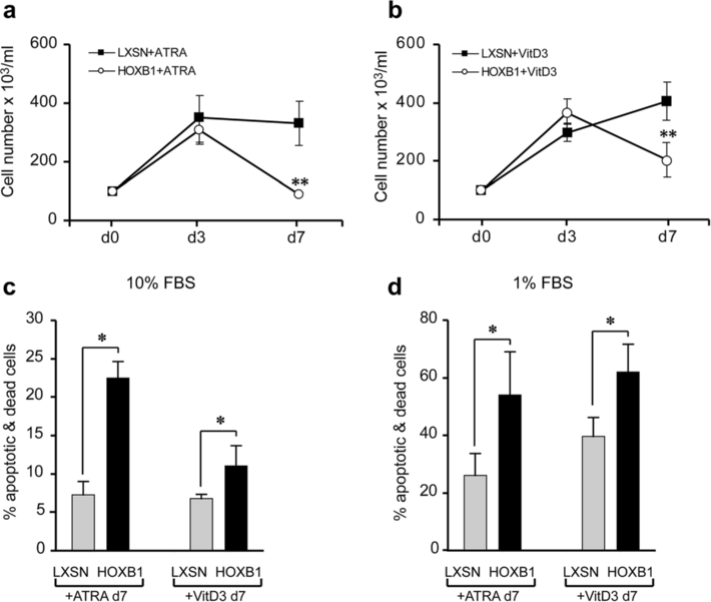
**Effects of HOXB1 restored expression on cell proliferation and apoptotic rates.** Cell growth curves in HOXB1- versus LXSN-transduced HL60 cells in ATRA (10^-7^ M) inducing granulocytic differentiation **(a)** and VitD3 (10^-8^ M) inducing monocytic differentiation **(b)**. Percentage of apoptotic & dead cells in HOXB1- versus LXSN-transduced HL60, in high **(c)** or low **(d)** serum conditions in presence of ATRA or VitD3. *p < 0.01; **p < 0.005.

The HOXB1-dependent effects in the HL60 cultures were then analyzed upon treatment with differentiating concentrations of all-trans-retinoic acid (10^-7^ M ATRA) or 1,25-dihydroxyvitamin D3 (10^-8^ M VitD3). Growth curves showed significant reductions of the HL60/HOXB1 cell growth respect to control cells in both culture conditions (Figure 3a and b). The percentage of apoptotic plus dead cells in 10% FBS cultures monitored for 7 days was almost doubled in HL60/HOXB1 cells treated with VitD3 (11% vs 6%) and three-fold more with ATRA (22% vs 7%) compared with LXSN corresponding controls (Figure 3c). In 1% serum the higher basal percentage of apoptotic plus dead cells observed in the LXSN controls was further enhanced by HOXB1, from 40% to 62% in VitD3- and from 26% to 54% in ATRA-treated cultures (Figure 3d).

### HOXB1 sensitizes HL60 to ATRA- and VitD3-induced differentiation

**Figure 4:**
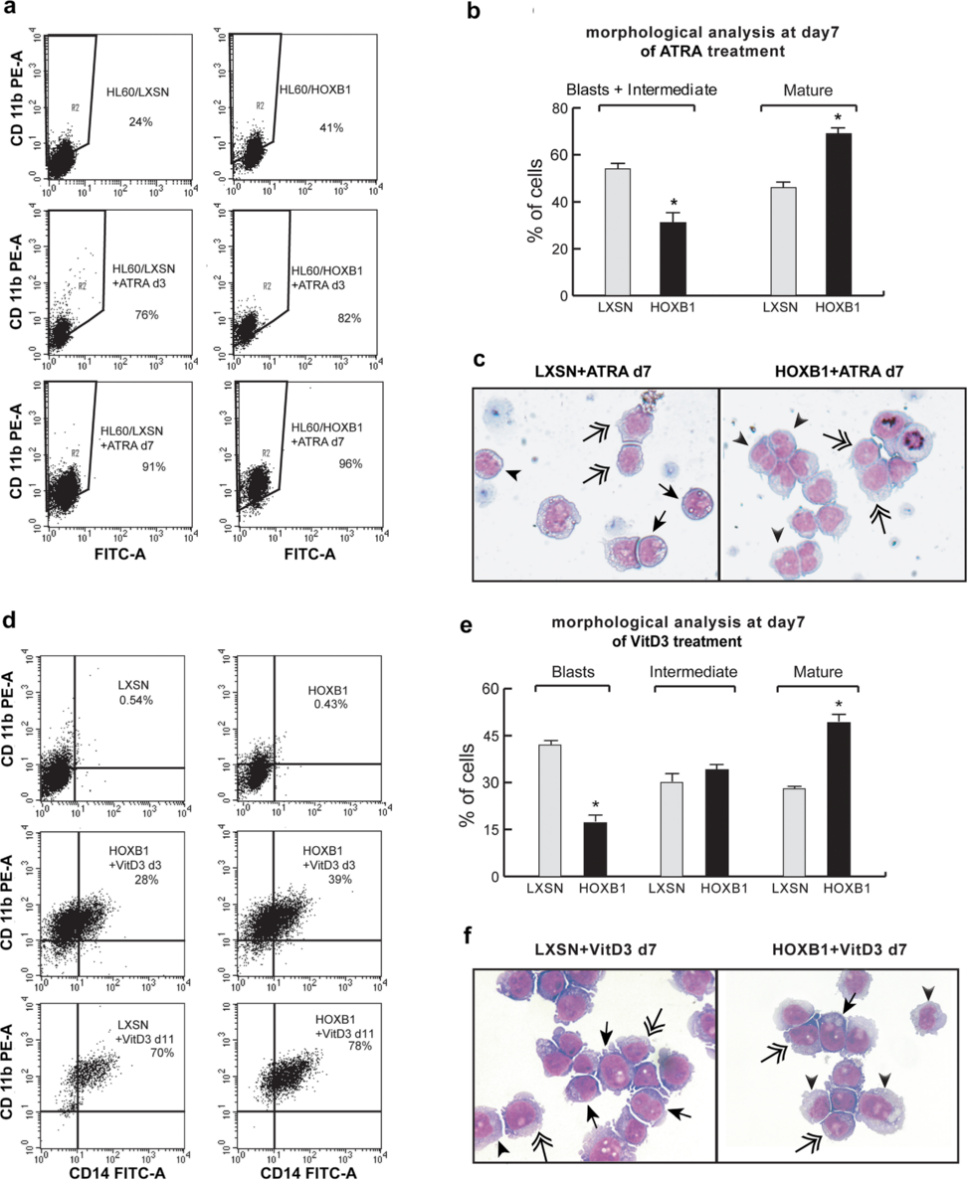
**Surface markers and morphological analysis in LXSN- and HOXB1-transduced HL60.** CD11b positive cells at day 0, 3 and 7 of ATRA (10^-7^ M) treatment **(a)**. Morphological analysis **(b)** and representative pictures **(c)** of ATRA-treated granulocytic-like cells. CD11b/CD14 double positive cells at day 0, 3 and 11 of VitD3 (10^-8^ M) treatment **(d)**. Morphological analysis **(e)** and representative pictures **(f)** of VitD3-treated monocytic-like cells. Cells were detected by the Giemsa-McGrünwald colorimetric method. Arrowheads indicate mature cells, long arrows blasts and double arrows intermediate cells.

We studied whether HOXB1 could have any effect on HL60 differentiation, alone or in synergy with the differentiating factors ATRA or VitD3. The onset of differentiation was estimated through a morphological analysis of the cells based on the Giemsa-McGrünwald colorimetric method, and the extent of differentiation was measured by FACS analysis of the cell surface markers CD11b, CD14 and G-CSFR. Although the percentage of CD11b positive cells was increased from 24 to 41% in LXSN- vs HOXB1-transduced cells, suggesting that HOXB1 per se might commit cells to granulocytic differentiation, the presence of HOXB1 did not seem sufficient to induce clear morphological changes during the myeloid maturation, at least in 10% serum (Figure 4a and data not shown). Nonetheless, after 7 days of ATRA treatment, although CD11b was highly expressed (>90%) in both HOXB1- and LXSN-transduced cells, the morphological analysis showed a higher number of terminally differentiated granulocytes (69% vs 46%) in HOXB1-transduced cells (Figure 4b and c). In the monocytic condition, the CD11b^+^/CD14^+^ markers associated with cell differentiation, showed 11% increase (from 28 to 39%) at day 3 and 8% (from 70 to 78%) at day 11 of culture in HOXB1- respect to LXSN-transduced cells (Figure 4d). Cell morphology showed a HOXB1 dependent increment in the number of terminally differentiated monocytes paralleled by a reduced amount of blast cells at day 7 (Figure 4e and f).

**Figure 5:**
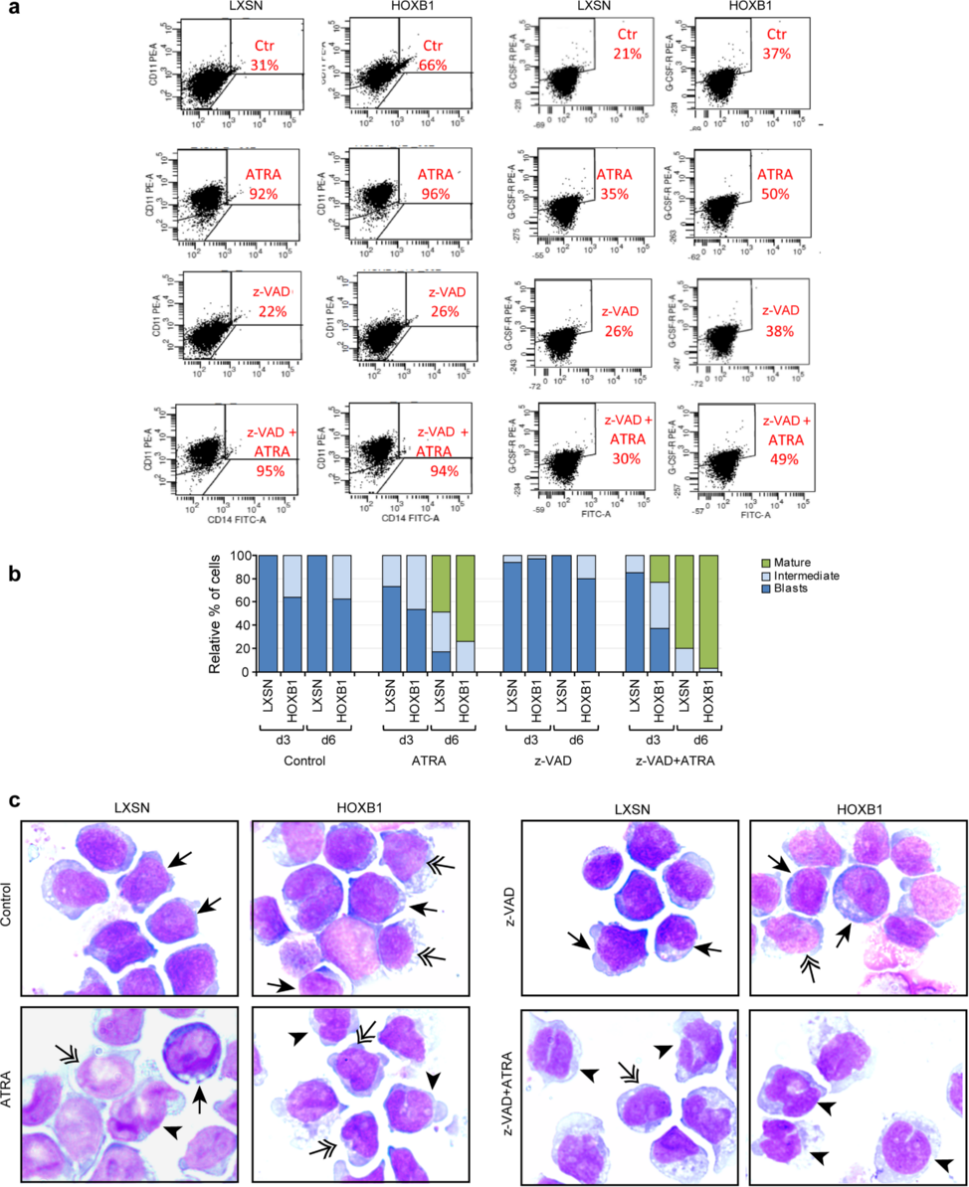
**Evaluation of ATRA-differentiation in presence of the apoptotic inhibitor z-VAD.** Surface markers analysis of CD11b (at day 6) and G-CSFR (after 1 hr) in LXSN- and HOXB1-transduced HL60 cells in low serum and ATRA (10^-7^ M), z-VAD (25 μM) and z-VAD (25 μM) + ATRA (10^-7^ M) treatments **(a)**. Morphological analysis **(b)** and representative pictures **(c)**. Cells were detected by the Giemsa-McGrünwald colorimetric method. Arrowheads indicate mature cells, long arrows blasts and double arrows intermediate cells.

Trying to understand the HOXB1-based mechanisms in inducing apoptosis and enhancing differentiation, we compared the differentiation level of HL60/HOXB1 vs control vector in presence or not of the caspase inhibitor z-VAD and 1% of serum. Firstly, in control conditions we confirmed the capability of HOXB1 to induce a certain degree of maturation (Figure 5a-c). Indeed, up to day 6 of cell culture, HL60/LXSN only included undifferentiated blasts, whereas approximately 40% of intermediate differentiated cells were detectable in HOXB1-expressing HL60 (Figure 5b). The percentage of CD11b and G-CSFR positive cells was increased from 31 to 66% and from 21 to 37% in LXSN- vs HOXB1-transduced cells, respectively (Figure 5a). As supported in terms of microscopic analyses and CD11b cell surface marker, the presence of z-VAD appeared to slightly interfere with the direct HOXB1 action. Conversely, the HOXB1-related differences, visible in ATRA-treated cells, were maintained by the combination with z-VAD, thus indicating that HOXB1-induced sensitivity to ATRA is maintained blocking apoptosis (Figure 5a-c). In these experiments the addition of z-VAD seemed to be even more effective on cell differentiation, possibly through an accumulation of mature cells otherwise addressed to death.

### Expression analysis of HOXB1-regulated genes

**Figure 6:**
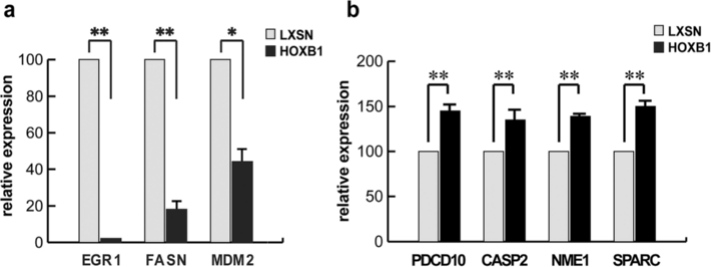
**Differential expressions in HOXB1- versus LXSN-transduced HL60 cells**. Real-time RT-PCR of EGR1, FASN and MDM2 downmodulated **(a)** or PDCD10, CASP2, NME1 and SPARC upmodulated **(b)** genes. GAPDH was used for normalization.*p < 0.01; **p < 0.005.

In order to gain insight in the molecular mechanisms underlying HOXB1 effects in the leukemic phenotype, we investigated genes differentially expressed in HOXB1-negative vs HOXB1-positive HL60 cells by probing an Atlas Human Cancer cDNA macroarray (Table 1). The expression level of some selected genes was confirmed by Real-time RT–PCR (Figure 6a and b). Interestingly, among the differentially expressed genes, we found molecules that could directly explain the reduced malignancy of HOXB1-transduced cells. Some tumour promoting genes, related to cell growth and survival, like the early growth response 1 (EGR1), the fatty acid synthase (FASN) and the mouse double minute 2 homolog (MDM2), resulted in fact strongly down-regulated (Table 1 and Figure 6a), whereas pro-apoptotic or tumor suppressor genes, as the caspase2 (CASP2), the programmed cell death 10 (PDCD10), the non metastatic cells 1 protein (NME1), and the secreted protein acidic and rich in cysteine (SPARC) were up-regulated (Table 1 and Figure 6b).

### HOXB1 promoter results methylated in HL60

**Figure 7:**
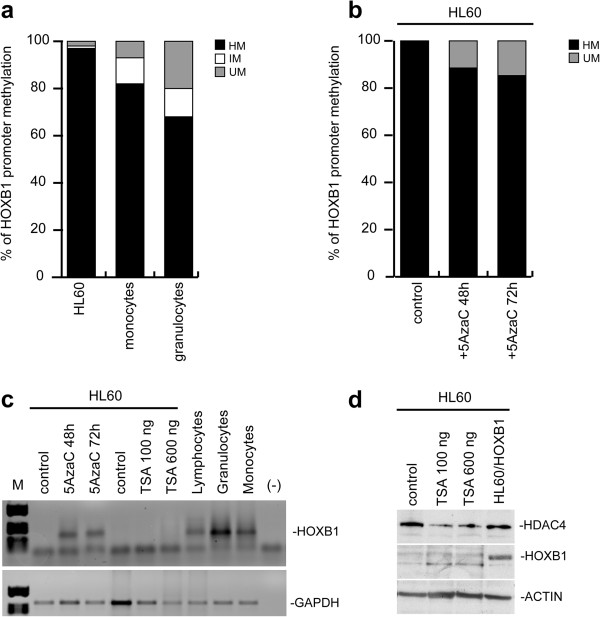
**HOXB1 promoter methylation in HL60 versus granulocytes and monocytes.** Relative percentage of DNA hypermethylated (HM), not methylated (UM), and intermediate methylated (IM) fractions **(a)**. HM and UM in 5-AzaC 1 μM treated HL60 cells after 48 h and 72 h **(b)**. RT-PCR evaluation of HOXB1 in 5-AzaC- and TSA-treated HL60 cells compared with lymphocytes, granulocytes and monocytes **(c)**. GAPDH is shown as quantitative standard. Western blotting of HDAC4 and HOXB1 in TSA-treated HL60 cells **(d)**. Actin was used as quantitative standard.

To investigate the possible mechanisms underlying HOXB1 downregulation in leukemic cells, we compared the methylation status of the CpG island present on HOXB1 promoter in HL60 and in normal monocytes and granulocytes from peripheral blood. As shown by three separate experiments, the hypermethylated (HM) fraction of the HOXB1 CpG island was significantly higher in HL60 respect to normal monocytes and granulocytes (97% respect to 82 and 68%) (Figure 7a). In order to verify the actual role of methylation on HOXB1 regulation, we treated the HL60 cell line with the demethylating drug 5-AzaC at 1 μM and 5 μM doses for 48 and 72 hrs. As the higher dose of 5-AzaC (5 μM) strongly reduced cell proliferation, we selected 1 μM dose for further studies. As expected, the HM fraction resulted decreased in 5-AzaC treated cells (Figure 7b) and its functional significance confirmed by re-expression of endogenous HOXB1 in the same samples (Figure 7c). On the contrary, we did not get any HOXB1 re-expression by treating the HL60 cells with the histone deacetylase inhibitor TSA (100 and 600 ng) for 8 hr and 24 hrs (Figure 7c and data not shown). As an internal control, the effectiveness of the TSA treatment was confirmed by the decrease of histone deacetylase 4 (HDAC4), one of the core components of the nucleosome (Figure 7d).

## Discussion

Numerous reports have catalogued differences in HOX genes expression between normal and neoplastic cells, but their functional relationship with the malignant phenotype in many cases remained elusive [22]. HOX genes are currently under evaluation in order to correlate specific HOX alterations with changes in cellular processes such as cell proliferation, differentiation and apoptosis. Other than HOX overexpression, also HOX downregulation has been associated with different malignancies, including leukemia. Examples of tumor suppressors are the homeodomain protein NKX3.1 and HOXD10 commonly down-regulated in human prostate cancer [23], breast tumor cells and gastric carcinogenesis [24,25]. In addition HOXA5 expression is lost in breast tumors [12] and HOXA genes, normally playing suppressor roles in leukemia development, are frequent targets for gene inactivation [26]. Accordingly, expression studies indicated a set of seven downregulated HOX genes (HOXA3, A4, A5, A7, B1, B9, C9) as significantly clustered in pediatric AMLs [11].

In this study we propose HOXB1 as an additional member of the HOX family with tumor suppressor properties. HOXB1 is expressed in terminally differentiated blood cells (erythrocytes, granulocytes, monocytes and lymphocytes) and in CD34+ progenitors from peripheral blood, but not in primary blasts from M1 to M5 and myeloid cell lines. Our results indicate a mechanism of CpG island promoter hypermethylation at the basis of HOXB1 silencing in AML as demonstrated by the higher amount of the hypermethylated DNA fraction in HL60 cells compared to normal cells. Accordingly, the demethylating agent 5-AzaC was able to reactivate HOXB1 expression in HL60 cells, whereas treatment with the histone deacetylase inhibitor TSA had no effect.

Results obtained by HOXB1 gene transduction in HL60, in agreement with the rapid counter-selection of the ectopic HOXB1 in AML193, U937 and NB4 cell lines (Additional file 1: Figure S1), point to the contribution of HOXB1 abnormal silencing to the survival of myeloid leukemic cells.

In HL60, HOXB1 restored expression was per se able to induce apoptosis and, in the presence of ATRA or VitD3, to favour maturation towards granulocytic and monocytic differentiation pathways, respectively. Of note, the HOXB1 induced differentiation, visible in ATRA-treated cells, does not appear associated with the apoptotic process, as shown by ATRA + z-VAD treatment.

According to our Atlas macroarray analysis, we identified a number of HOXB1 dependent up- and down-modulated genes. Specifically, we observed the up-regulation of some apoptosis-related genes as CASP2, JNK2, PDCD10, SPARC and heat-shock protein 70 kD-interacting protein (ST13). In particular CASP2, JNK2, PDCD10, and ST13 have been associated with mitochondrial permeabilization [27-30] and with the induction of the apoptotic process, while SPARC overexpression seems to play a tumor suppressor function in some low expressing SPARC AMLs [31,32]. As in HOXB1-transduced cells we also observed a significant enhancement of APAF1 (Figure 2e), we suggest the involvement of HOXB1 in triggering the mitochondrial as well as caspase dependent apoptotic pathways [33], as indicated by the activation of caspase 3/7 (Figure 2d,e). Accordingly we also detected a HOXB1-dependent regulation of the BCL-2 family of proteins playing a major role in the control of apoptosis. In particular, the proapoptotic role of HOXB1 was sustained by the induction of BAX and the downregulation of MCL1 proteins. Moreover the BAX/BCL2 ratio, doubled by HOXB1, was indicative to increased cell susceptibility to apoptosis [34]*.*

In addition, the macroarray analysis showed the HOXB1-dependent downregulation of some antiapoptotic genes as MDM2, FASN, the antioxidant enzyme superoxidedismutase (SOD1) and the breast cancer susceptibility gene 2 (BRCA2). As the knockdown of MDM2 in p53 mutant non-small cell lung cancer [35,36], the FASN reduced expression in HepG2 cells [37,38] or the SOD1 downregulation in AMLs [39,40] can induce apoptosis, we might suggest a HOXB1 related anticancer activity. Nonetheless, as p53 is not expressed in HL60 cells, we should consider the involvement of other members of the p53 family, as p63 and p73 expressed in HL60 cells [41]. Specifically p63 has been described to be activated by PBX cofactors [42] and in HL60 cells we observed a HOXB1-related induction of PBX2 (data not shown), thus possibly suggesting the effectiveness of p63 downstream to HOXB1.

Finally, EGR1 displayed a striking downregulation. Although deserving further studies due to its complex and somehow divergent activities, its reduction was in agreement with the lower tumorigenicity of HL60 cells overexpressing HOXB1. In fact EGR1 has been reported to play a role in prostate tumor growth and survival [43] and its abnormal expression has been recently associated with tumor invasion and metastasis in gastric cancer [44]. In addition, a higher level of EGR1 has been associated with relapsing AML respect to AML at diagnosis with a direct correlation with increased proliferation and enhanced RAF/MEK/ERK1/2 activation [45].

In conclusion our results indicate an antineoplastic role for HOXB1 in AMLs through its functional involvement in promoting apoptosis and powering ATRA-induced differentiation. Considering the presence of two RARE elements at the 5′ and 3′ ends of HOXB1 [46], we might suggest a role for HOXB1 in ATRA-mediated anticancer activity. In this view a HOXB1/ATRA combination might represent a possible future therapeutic strategy in AML [47,48].

## Consent

Informed consent for publication was obtained from the patients in accordance with the Declaration of Helsinki.

## Competing interests

The authors declare that they have no competing interests.

## Authors’ contributions

Conceived and designed the experiments: MP, FF and AC. Performed the experiments MP, FF, LB, MCE, OM, AB and ADF. Wrote the paper: MP, FF and AC. All authors read and approved the final manuscript.

## Supplementary Material

Additional file 1: Figure S1 Effects of HOXB1 restored expression in U937 and NB4 cell lines. Click here for file
