# Supplementary material for: HOXB1 restored expression promotes apoptosis and differentiation in the HL60 leukemic cell line
Source: Cancer Cell Int. 2013 Oct 22;13:101. doi: 10.1186/1475-2867-13-101 (PMC3874656; doi:10.1186/1475-2867-13-101)
Supplement: Additional file 1: Figure S1 — Effects of HOXB1 restored expression in U937 and NB4 cell lines. [file 1475-2867-13-101-S1.pdf]

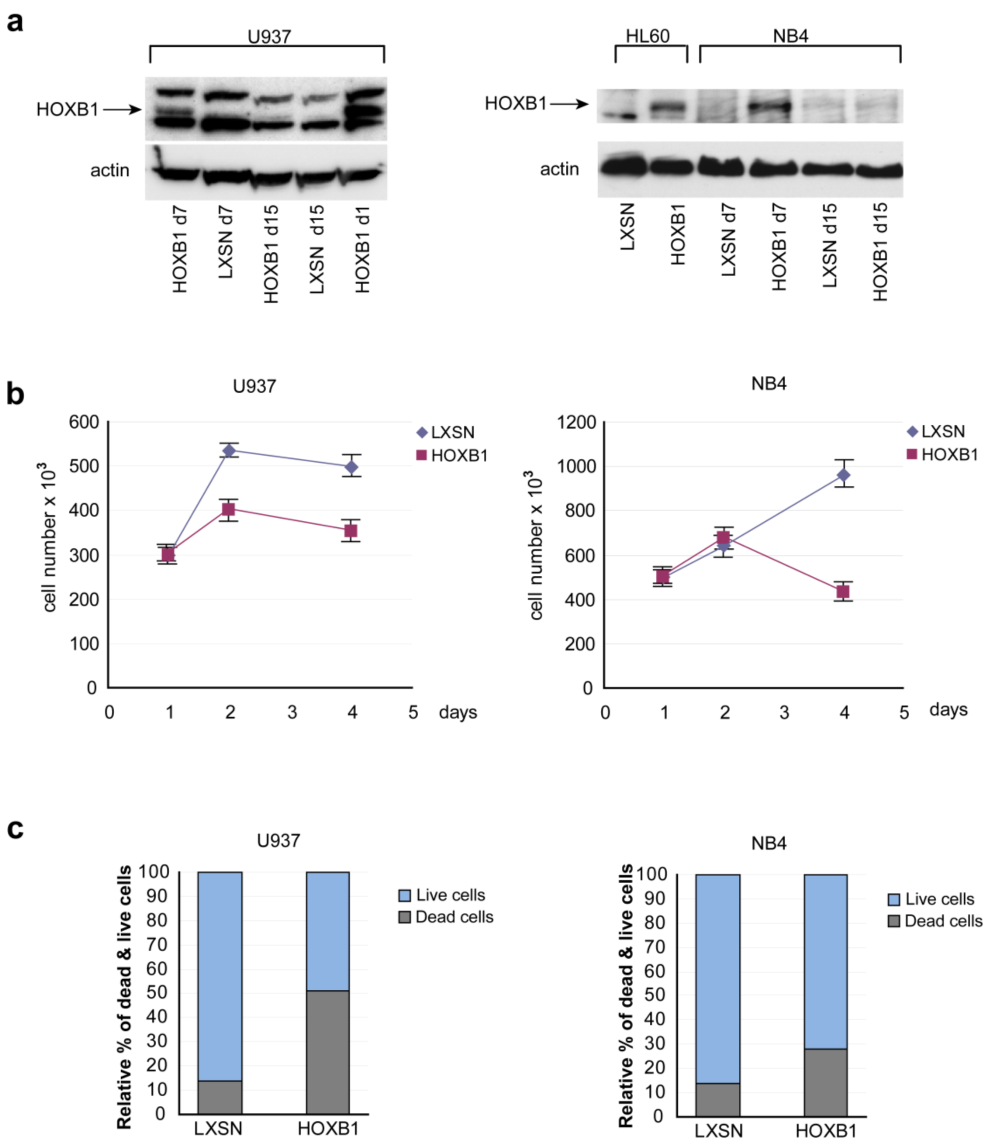

Supplementary Figure 1

Effects of HOXB1 restored expression in U937 and NB4 cell lines.
Western blot analysis of HOXB1 in neomycin selected LXSN- and HOXB1-transduced U937 and NB4 cell lines. Actin was used for normalization (a). Analysis of cell growth (b) and relative percentage of live and dead cells (c) in low serum condition; *p<0.01.
